# Orchestration of Neutrophil Extracellular Traps (Nets), a Unique Innate Immune Function during Chronic Obstructive Pulmonary Disease (COPD) Development

**DOI:** 10.3390/biomedicines9010053

**Published:** 2021-01-08

**Authors:** Anjali Trivedi, Meraj A. Khan, Geetanjali Bade, Anjana Talwar

**Affiliations:** 1 Department of Physiology, All India Institute of Medical Sciences, New Delhi 110029, India; anjali.trivedi12@gmail.com (A.T.); geetanjalibade@gmail.com (G.B.); 2 Translational Medicine, SickKids Research Institute, the Hospital for Sick Children, Toronto, ON M5G 1X8, Canada

**Keywords:** chronic obstructive pulmonary disease (COPD), pathogenesis, innate immunity neutrophils, neutrophil extracellular traps (NETs), NETosis, NOX-dependent and NOX-independent

## Abstract

Morbidity, mortality and economic burden caused by chronic obstructive pulmonary disease (COPD) is a significant global concern. Surprisingly, COPD is already the third leading cause of death worldwide, something that WHO had not predicted to occur until 2030. It is characterized by persistent respiratory symptoms and airway limitation due to airway and/or alveolar abnormalities usually caused by significant exposure to noxious particles of gases. Neutrophil is one of the key infiltrated innate immune cells in the lung during the pathogenesis of COPD. Neutrophils during pathogenic attack or injury decide to undergo for a suicidal death by releasing decondensed chromatin entangled with antimicrobial peptides to trap and ensnare pathogens. Casting neutrophil extracellular traps (NETs) has been widely demonstrated to be an effective mechanism against invading microorganisms thus controlling overwhelming infections. However, aberrant and massive NETs formation has been reported in several pulmonary diseases, including chronic obstructive pulmonary disease. Moreover, NETs can directly induce epithelial and endothelial cell death resulting in impairing pulmonary function and accelerating the progression of the disease. Therefore, understanding the regulatory mechanism of NET formation is the need of the hour in order to use NETs for beneficial purpose and controlling their involvement in disease exacerbation. For example, DNA neutralization of NET proteins using protease inhibitors and disintegration with recombinant human DNase would be helpful in controlling excess NETs. Targeting CXC chemokine receptor 2 (*CXCR2*) would also reduce neutrophilic inflammation, mucus production and neutrophil-proteinase mediated tissue destruction in lung. In this review, we discuss the interplay of NETs in the development and pathophysiology of COPD and how these NETs associated therapies could be leveraged to disrupt NETopathic inflammation as observed in COPD, for better management of the disease.

## 1. Introduction

Chronic obstructive pulmonary disease (COPD) is a progressive respiratory disease and is one of the leading cause of morbidities and mortality throughout the globe. The Global Initiative for Chronic Obstructive Lung Disease (GOLD) committee defines COPD as a common, preventable and treatable disease [1]. The airflow limitation is usually progressive, not fully reversible and associated with abnormal inflammatory response of the lungs. According to the Global Burden of Disease (GBD), COPD is already reached as the third leading cause of death globally. This is something, WHO had not predicted to occur until 2030 [2]. Persistent airflow limitation, emphysematous alveolar wall destruction, increased persistent neutrophil infiltration and recurrent infections are the major distinctive features of COPD [3]. Neutrophil is one of the key infiltrated innate immune cells in the lung during the pathogenesis of COPD [4,5]. The release of chemotactic factors secreted by structural cells (e.g., epithelial cells) and by resident inflammatory cells (e.g., macrophages) contribute to the neutrophilic increased influx and inflammation in the lungs [6]. The bronchoalveolar lavage fluid (BALF) analyses of the COPD patients, show the presence of CXC chemokines, including *CXCL1* (*GRO-α*), *CXCL5* (*ENA78*), leukotriene B4 (*LTB4*) and chemokine (C-X-C motif) ligand 8 or interleukin-8 (*CXCL8*) are the major neutrophilic chemoattractant [7]. Neutrophil acts as a first line of immune defense by reaching first to the site of action, confining pathogens and resolving infections. Thus, optimal physiological regulation of neutrophil mediated immunity is needed for an effective host-defense. However, dysfunction of these cells and/ or process is associated with bystander effect in immune response [8].

Recently, a unique form of neutrophil cell death discovered during the infection or injury, that involves the formation of Neutrophil Extracellular Traps (NETs). These unique DNA-entangled protein mesh capture and ensnare the pathogens [9]. The whole process of the formation of NETs, also known as “NETosis”, is different from apoptosis and necrosis [10,11]. This restricts the potential pathogen dissemination from the initial site of infection and allows a complete neutrophil microbicidal function through a series of activated signaling pathways [12]. It’s the tissue microenvironments that determine to opt either phagocytosis or the release of NETs. However, NETs can be detrimental too to the surrounding tissue, depending on the location, timing, and extent of inflammatory response [13]. Therefore, too much of NETs at a particular time or location can cause tissue damage of the host organism and may associate with many pathological conditions [14]. Based on many reports and published data, NETs considered as a “double edged” sword in innate immunity. Collectively, the good/bad side aspect dependents on maintaining a tight equilibrium between protective and detrimental immune responses [15]. NETs function as a valuable antimicrobial defense mechanism as they can entrap and kill pathogens [14]. On the other hand, NETs are associated with many pathological situations including autoimmune diseases, infection, sepsis, lung damage, cancer metastasis, thrombosis, and fibrosis and even in COVID-19 lungs that causes ongoing pandemics [4,16,17,18,19,20]. NETs promote the activation of lung fibroblasts and their differentiation into myofibroblast phenotype [21]. The expression of myofibroblast in alveoli and airways is also affected by smoking and COPD [22] which increases small airway thickening and decreases lung function in COPD. Neutrophil Elastase also promotes myofibroblast differentiation in lung fibrosis [23].

Therefore, it is of great clinical significance to acknowledge both beneficial and detrimental effects of NETs to understand the regulatory mechanisms of the NETs and to devise some therapeutics options for the clinical management of associated disease conditions.

The presence of NETs in the airways of patients with COPD, and asthma have been demonstrated by microscopic studies [24,25]. Increased quantities of NETs and NET producing neutrophils are observed in sputum samples of stable COPD patients and during exacerbations [25]. The triggers for NET formation include proinflammatory cytokines- *CXCL8* and Tumor Necrosis Factor α (*TNF-α*), activated platelets, bacterial products (formylated peptides and lipopolysaccharides (LPS), fungi, bacteria (Pseudomonas aeruginosa and Hemophilus influenzae), and immunoglobulins [26,27,28,29]. Most of these proposed drivers of NET formation are present in the airways of COPD patients under stable conditions, thus evaluating the drivers of NET formation in COPD patients is challenging [25]. Previous studies indicated that CXC chemokine receptor 2 (*CXCR2*) antagonists can reduce neutrophils in the lungs of patients with COPD and thus can limit the harmful effects of neutrophils on the lung tissue [30].

Thus, in this review, we discuss the interplay of NETs, a unique function of innate immune cells in the development and pathophysiology of COPD. NETs being considered as double-edged sword. It is important to understand and highlight how these NETs associated therapies could be leveraged to disrupt NETopathic inflammation as observed in COPD, to better manage the disease.

## 2. Chronic Obstructive Pulmonary Disease (COPD)

COPD is a heterogeneous disease displaying varying patterns of airway inflammation, parenchymal destruction and lung function decline, thereby resulting in numerous pathobiological trajectories [31,32]. Approximately 75% of COPD cases are attributed to cigarette smoking [33]. Besides cigarette smoking, an additional risk factor for COPD is occupational exposure to fumes, gases, dusts and vapors which may account for another 15% of COPD cases. Other factors including genetic factors, respiratory infections, indoor and outdoor exposure to air-pollutants also play role in COPD development and progression [34]. Understanding the pathophysiology of the disease is important to review the involvement of innate immune cells during the disease progression.

### 2.1. Pathophysiology of COPD

Noxious agents in the cigarette smoke injure the airway epithelium and activate the inflammatory cells to release a combination of proteases and inactivate several anti-proteases, resulting in protease and anti-protease imbalance [35,36]. Increased oxidative stress and protease to anti-protease imbalance are the other major phenomena involved in disease initiation and progression [37]. The hallmarks of smoking induced COPD include squamous cell metaplasia and goblet cell hyperplasia which causes increased cough and mucus secretion [38]. Mucociliary clearance is disrupted by the bronchi undergoing squamous metaplasia [39] and situation collectively leads the persistent airway inflammation.

### 2.2. Inflammation in COPD

**Figure 1:**
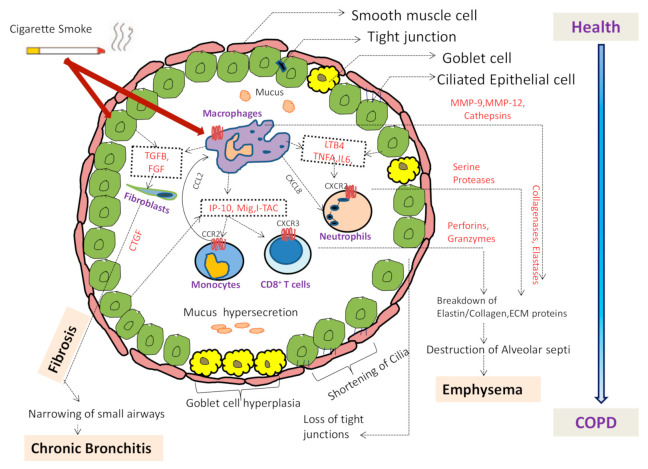
Airway illustration showing the pathological changes in disease condition. Cigarette smoke activates macrophages and epithelial cells in the respiratory tract to release various chemotactic factors. Various cells attracted by these chemokines and cytokines including neutrophils by *CXCL8*, monocytes by *CCL2* and T cells by *CXCL12*. Furthermore, the activation of neutrophils, monocytes, T cells, fibroblasts and airway smooth muscle leads to release of many more cytokines and chemokines. Epithelial cells and macrophages also release fibrogenic mediators like transforming growth factor beta (*TGFB*) and fibroblast growth factor (*FGF*) that activates fibroblasts and release connective tissue growth factor (*CTGF*) resulting in fibrosis of small airways. Proteases, MMPs, cathepsins, collagenases and elastases released by neutrophils and macrophages leads to breakdown of extracellular matrix (ECM) leading to emphysema. Shortening of cilia, loss of tight junctions and goblet cell hyperplasia leading to mucus hyper secretion are other changes occurring in COPD.

COPD is pathologically mediated by many inflammatory pathways [40]. The illustration Figure 1 shows the complex immune and inflammatory process occurring in the airway lumen of the patients during disease development and progression. Both innate and adaptive immune responses are involved in the inflammatory response of COPD [41]. The specific inflammatory pattern observed in the lung parenchyma and airways of COPD patients, consists of increased number of inflammatory cells like macrophages, neutrophils, natural killer (NK) cells and T-lymphocytes [42,43]. Interestingly, studies have also reported presence of eosinophils in the airways, tissues, and circulation in COPD patients during both stable disease and exacerbations [44]. Airway epithelial cells and the surface macrophages are activated by the cigarette smoke and other irritants, which then triggers release of chemokine factors such as *TNFα*, Interleukin 1 beta (*IL-1β*), Granulocyte-macrophage colony-stimulating factor (*GM-CSF*), *CXCL-8*. The circulating leukocytes are attracted into the lungs by the released chemokines [43]. Macrophages activate other inflammatory cells to release chemotactic and fibrogenic factors such as connective tissue growth factor (*CTGF*) and *TGFβ* [35]. There is increased recruitment of monocytes and T lymphocytes from the circulation in response to monocyte-selective chemokines and lymphocyte chemotactic factors respectively. These cells release inflammatory mediators, when activated by cigarette smoke extract, thus providing a cellular mechanism that links smoking with inflammation in COPD [45].

Neutrophils releases reactive oxygen species (ROS) and Serine proteases like neutrophil elastase (NE), cathepsin G, and proteinase-3, as well as matrix metalloproteinase (MMP)- *MMP-8* and *MMP-9*, which causes alveolar destruction [46]. These serine proteases are also potent mucus stimulants. Host tissue damage is also caused by neutrophils which lead to initiation of inflammatory response thus causing recruitment and activation of other inflammatory cells [47]. Increased number of inactive eosinophils has been found in airways and lavage of COPD patients while some studies have reported no evidence of increased eosinophils in BAL or induced sputum [48,49]. T-lymphocytes are also increased in lungs as well as airways of COPD patients [50]. The number of CD8^+^ T cells is greater than that of CD4^+^ cells and the ratio of CD4^+^/CD8^+^ cells is reversed in COPD [51]. Perforins and granzymes are released by the CD8^+^ cells, which also can cause cytolysis and apoptosis of the alveolar epithelial cells [52]. Complement anaphylatoxins (C3a and C5a) are potent inflammatory peptides involved in exaggerated inflammatory responses observed in COPD exacerbation [53]. *C5a* induces activation of *NF-KB* thus enhancing production of various cytokines. In vivo results showed that local and systemic C5a concentrations increased in acute exacerbations of COPD. Animal models have also reported that *C3a* and *C5a* can promote many pathogenic features of COPD like smooth muscle contraction, enhancement of airway hyper responsiveness and vasodilation in lungs [54].

### 2.3. Adverse Effect of Cigarette Smoke via Neutrophils

Along with the long-term lung irritants exposure to the lungs, cigarette smoking is one of the key factors that contribute COPD disease progression. Smoking and second-hand smoke exposure during childhood and teenage years can slow lung growth and development. In a nutshell, this can increase the risk of developing COPD in adulthood. At molecular level, cigarette smoke extract (CSE) causes degranulation of secondary granules from neutrophils thus contributing to airway inflammation and tissue degradation [14,55]. Furthermore, the ability of ingesting respiratory pathogens is seen to be compromised in CSE exposed neutrophils. Thus, it leads to persistent existence of bacterium in smoker’s lung and promotes further neutrophil recruitment [55]. This situation leads the overactive immune cells recruitment response in the lung. After exposure to CSE, human neutrophils share typical cell death features such as apoptosis, autophagy and necrosis. Neutrophils could be recognized and phagocytized by macrophages [14,55]. It can also undergo a spontaneous and phagocytosis induced apoptosis in *caspase-3* dependent manner. CSE suppresses the *caspase-3* activity and does not alter spontaneous apoptosis but impairs the phagocytic activity [56]. The percentage of sputum neutrophils undergoing spontaneous apoptosis is reduced significantly in COPD patients. It also leads to persistent existence of neutrophils in smokers lungs [56]. The degree of neutrophilia correlates with COPD severity, exacerbations, and disease progression [25]. Neutrophil chemotaxis, neutrophil extracellular trap formation and inflammatory response-related gene expression is modified by cigarette smoke [57].

CS could induce necrotic neutrophil cell death through mitochondrial dysfunction, apoptosis inhibition and damage associated molecular pattern (DAMP) release as shown by in vitro experiments [58]. During COPD exacerbations, DAMP signaling plays role in activation of neutrophils. DAMPs can activate the innate immunity by binding to Pattern recognition receptors (PRRs) such as *TLR2*, *TLR4* and *TLR9*. Serum levels of DAMP gene expression are increased during COPD exacerbations [59]. Elevated airway inflammation is observed during COPD exacerbations due to activation and migration of neutrophils caused due to activation of *TLR2/TLR4*. CSE triggers the release of NETs that subsequently induces fibroblasts activation/differentiation [60].

Cigarette smoke –induced autophagy impairment accelerates lung-aging, COPD exacerbations and pathogenesis [61]. Lungs from old mice showed accumulation of aggresomal bodies. Increase in levels of aggresomal bodies in lungs of COPD patients was observed by Vij et al. (2018) [61]. Increased autophagy contributes to COPD pathogenesis by promoting epithelial cell death. Increased autophagy in clinical specimen of lung tissue from COPD patients and increased expression and activation of autophagic regulator proteins (LC3B, Beclin 1, Atg 5 and Atg 7) was observed [62]. *Egr-1* plays a critical role in promoting autophagy and apoptosis in response to CSE in vivo and in vitro. Yoshida et al. demonstrated the involvement of CS induced epithelial cell ferroptosis in pathogenesis of COPD [63].

## 3. Innate Immune Cell Neutrophils and COPD

Neutrophil plays a key role in antimicrobial defence in COPD patient’s airway mucosa as it contains proteases, inflammatory mediators and oxidants. *IL-22/IL-22R* signaling pathways plays role in antimicrobial defense [64]. At the same time Neutrophil derived proteases impairs the antimicrobial *IL-22/IL-22R* signaling pathways and decreases the expression of antimicrobial effectors such as β-defensin-2 which in turn enhances the pathogen replication and leads to COPD exacerbations [14,64].

COPD patients, excess neutrophils are recruited to the airways and their proteases such as neutrophil elastase (*NE*), myeloperoxidases (*MPO*) cause alveolar tissue destruction [65]. The severity of symptoms in COPD is directly correlated with the activation of neutrophils in the lungs [66]. Sputum Neutrophil percentage was directly correlated with dyspnea scores across different severity of COPD [67] and with poor prognosis.

In COPD patients, exposure to bacterial pathogens can cause innate immune responses in neutrophils thus leading to increased expression of *CXCL-8*, *TNF-α*, Interferon gamma (*IFN-ϒ*) and Interleukin 6 (*IL-6*) [14,68]. Neutrophils from COPD patients are impaired in function as they demonstrate migratory inaccuracy. Previous reports indicate contradictory data regarding the phagocytic functions of neutrophils in COPD [5,14]. Some studies show reduced ingestion of opsonized species [69,70]. While other studies suggest no difference between the phagocytic abilities of COPD neutrophils and controls [71,72].

### 3.1. Neutrophils: Link between Innate and Adaptive Immunity

Neutrophils are one of the important components of immune defence barrier linking innate and adaptive immunity. Traditionally neutrophils are considered as an innate immune cell [73]. Being an essential component of innate immunity, neutrophils were involved in killing of pathogens and removal of cellular debris by phagocytosis and/or degranulation or release of neutrophil extracellular traps (NETs) [74].

Neutrophils are important orchestrators of adaptive immunity. They cross talk with lymphocytes and Antigen presenting cells either directly via cell-cell contact or via mediators such as cytokines and chemokines [75]. Neutrophils can also acquire features of APC under the inflammatory microenvironment and can lead to activation of adaptive immunity [76]. Neutrophils can recruit T lymphocytes to inflammatory sites and activate them [77]. Neutrophils are important mediators of TH17 cells-controlled pathway of resistance to pathogens. The cytokines secreted by Th17 cells such as Interleukin 17 (IL-17), CXCL8, IFN-ϒ, TNF-α and GM-CSF promotes granulopoiesis and favors recruitment, activation and prolonged survival of neutrophils at the site of inflammation [78]. Neutrophils are crucial for development of NK cells and a bidirectional cross talk between these cells stimulates IFN-ϒ production by NK cells and which promotes the activation and survival of neutrophils [79]. A novel link between neutrophils and adaptive immune responses is demonstrated by NETs mediated T cell interaction. NETs produced by neutrophils impact adaptive immunity by influencing dendritic cell maturation [76].

### 3.2. Neutrophil Migration

Neutrophils migration from one organ to other or infiltration at the site of action is one of the important aspects of its functionality. In the lung, neutrophils are usually recruited from the circulation to the airways of COPD patients [80]. As evident from sputum and blood analysis, the principal signals for neutrophil influx in COPD airways are LTB4, CXCL-8 and Interleukin 10 (IL-10) [3]. Other chemotactic factors include C5a, CXCL1, CXCL5 and elastase-α1-antitrypsin complexes [81]. In COPD patients, neutrophils are recruited to the airways and serine proteases such as Neutrophil Elastase (NE), Myeloperoxidases (MPO) are secreted by these cells which lead to alveolar tissue destruction [82,83]. The structural components of Extracellular matrix (ECM) are degraded by NE which is a neutrophil derived serine proteinase and has a role to play in tissue damage and remodeling [83]. NE causes elastin breakdown which in turn is associated with COPD-induced inflammation [84]. Thus, NE causes fibroblast proliferation, matrix degradation and mucus metaplasia and all these combined effects of NE accelerate the small airway obstruction in COPD. A-1-Antitrypsin (*A1AT*) is an endogenous inhibitor of NE and can limit lung damage [85]. MPO is an inflammatory mediator which is mainly stored in the primary granules of neutrophils and is upregulated during the inflammatory response. It is also responsible for accelerating the inflammatory response [86]. Matrix metalloproteases (MMPs) are zinc dependent proteases which are secreted by Neutrophils and macrophages. MMP-1, MMP-9 and MMP-12 are mainly implicated in emphysema pathogenesis [87,88]. Mature Neutrophils synthesizes MMP-9 and it contributes to airway obstruction by destroying the structural components of ECM as shown by increased MMPs in Bronchoalveolar lavage fluid (BALF) and plasma of emphysema patients [66]. Activation of signaling pathways occurs followed by cytoskeletal rearrangements and changes in cell surface molecules which coordinate to facilitate neutrophil migration [65,81].

### 3.3. Functions of Neutrophil and Death Mechanisms

Bactericidal functions are performed by neutrophils in three ways, including phagocytosis (releasing bactericidal granular proteins, reactive oxygen species, and reactive nitrogen species (RNS), degranulation and by forming NETs to trap and ensnare [12,66,89,90,91].

#### 3.3.1. Neutrophil Phagocytosis and Degranulation

The micro-organism is engulfed by the neutrophils (phagocytose) following the formation of phagosome that go through a series of changes known as phagosome maturation to make it suitable for pathogen killing [92]. A dynamic process of sequential events collectively brings many changes to the contents and the membrane of the phagosome [74]. Microbicidal enzymes, vacuolar ATPases and NADPH oxidase complex are involved in the formation of phagosomes [66]. Sequential fusion with early and late endosome and finally with lysosomes occurs thus yielding a phagolysosome. Microbial peptides and proteolytic enzymes are present in the vesicles of neutrophils. Four types of granules are named after the order of development. Primary granules (azurophilic granules) contain MPO and membrane bound sialoglycoprotein (CD43). Secondary granules (specific granules) contain lactoferrin and membrane bound carcinoembryonic antigen related cell adhesion molecule-8 (CD66b). Tertiary granules contain gelatinase. Fourthly, secretory vesicles contain albumin and expresses alkaline phosphatase and complement receptor type-1 (CD35) for C3b/C4b- coated particles on their membrane [93]. At high cytosolic calcium levels, annexins mediates the fusion of phagosome with the granules [94]. Phagosome contains granular proteins and also ROS and RNS, thus the total protein mix present in phagosomes is different from that present in all the granules. After granular fusion, the granular contents start their work in killing pathogens [66,92]. TLRs play an important role in this process. Upon binding to the membrane proteins on gram positive bacteria, *TLR2* triggers phagocytosis, whereas *TLR4* does the same for gram negative bacteria.

Neutrophils release a mixture of proteins in three types of granules by a process called degranulation. Vesicles of neutrophils contain many adhesion molecules and receptors [95]. The priming mediated fusion of the secretory granules with the plasma membrane leads to augmentation of the adhesion capacity and the activation potential. Thus, the fixed order of granule fusion with the phagosome starts with secretory vesicles followed by gelatinase granules, specific granules and ends with the azurophilic granules due to their different calcium thresholds for secretion [96]. Report by Koenderman et al. suggests that circulating neutrophils from COPD patients are primed and this priming is particularly noted during exacerbations [97,98].

#### 3.3.2. Apoptosis of Neutrophils

Average life of neutrophil ranges from hours to few days. This is terminally differentiated cells and most of the cell machinery support quick functionality and that helps to respond against any pathogen attack or injury. Activated neutrophils rapidly undergo apoptosis [99]. A series of characteristic morphological changes such as membrane blobbing, cell body shrinkage, cytoplasm densification, condensation of nuclear chromatin and cutting of genomic DNA by endonucleases occurs [100,101,102,103]. Thus, apoptosis helps to minimize any permanent damage which may be caused due to inflammation. Neutrophil apoptosis involves participation of two main pathways: The extrinsic (death receptor) pathway which occurs as a result of surface death receptors that bind to TNF-related apoptosis-inducing ligand (*TRAIL*), *TNFα* or Fas ligand (*FasL*) [104]. The Intrinsic pathway is directly linked to the participation of mitochondria [103]. Raised levels of GM-CSF, CXCL-8 and LTB4 delays neutrophil apoptosis in COPD patients. Decreased muco-ciliary clearance in COPD also leads to longer retention of apoptotic neutrophils [7].

### 3.4. Neutrophils Decision to Phagocytosis or Formation of NETs

In order to efficiently clear pathogens and minimize host damage, neutrophils possess the capacity to make important decisions that define the antimicrobial strategies they undertake after being recruited to the site of inflammation. The choice of neutrophils to either phagocytose or generate NETs is influenced by many factors which includes the environmental conditions, the activation, adhesive and metabolic state of phagocyte [27,90,105,106,107,108,109,110,111,112,113,114,115]. Besides this, the size and signals associated with the tethered phagocytic cargo also influences neutrophil’s choice. The major factors that determine the decision of neutrophils to phagocytose or form NETs are represented in Table 1. Thus, we have seen that several factors regulate the phagocytosis and generation of NETs, prompting one event to negatively regulate the other. The key factors responsible for neutrophil’s fate to go do phagocytosis or to form NETs are enlisted in Table 1.

## 4. Neutrophil Extracellular Traps (NETs)

Takei et al. first observed that neutrophils released chromatin-containing content when activated by Phorbol-myristate-acetate (PMA) [118]. This form of cell-death was different from apoptosis and necrosis. It is a cell death pathway whose principal consequence is extracellular traps (ETs) formation. The formation of neutrophil extracellular trap (NETs), a unique regulatory process of neutrophils in response to pathogens or injury unlike phagocytosis and apoptosis, was formerly reported in 2004 [11,26]. These NETs are characterized to trap and kill different pathogens including virus, bacteria, fungi and many more pathogens [26,91,119,120]. The large web like structures consists of decondensed chromatin studded with several granules and nuclear proteins [26,121]. These decondensed DNA entangled with many antimicrobial peptides, helps in trapping and killing the pathogens. The whole process of formation of NETs is termed as NETosis. Some reports have classified the NETosis as vital and suicidal based on the fate of neutrophils.

Though NETosis was first described in neutrophils, other cell types, such as eosinophils, mast cells, monocytes and macrophages, are also capable of releasing ETs composed of DNA and antimicrobial proteins. This mechanism causes death of these cells. These cells can also cause death by this mechanism hence the process was renamed as ETosis, which refers to cell death with the release of ETs [120,122,123,124,125,126,127]. The major mediators and components of NETs are represented in Table 2. As compared to protein component, the DNA constituents present in NETs are of utmost significance in maintaining the composition of NETs and acts as backbone of the unique mesh structure [26]. This is evident from studies reported by Von Köckritz-Blickwede et al. in which treatment with DNase enzyme leads to its degradation while the structural integrity is maintained when treated with protease enzymes [128,129]. The anti-microbial activities of NETs are dependent on the proteinaceous components of NETs [91]. H3 and H4 histones were shown to aggregate in their structure type A influenza virus while H1 binds some noroviruses. Recent research showed that some NET components, including myeloperoxidase, cathelicidin and α-defensins display strong antiviral properties. α-defensins show their biocidal activity both in enveloped and non-enveloped viruses [130]. The glycoprotein lactoferrin chelates iron and calprotectin sequesters zinc ions. Calprotectin was found to be the crucial protein involved in NET degradation of fungi [131]. The post translational modified proteins that constitutes NETs can become target auto-antigens contributing to auto-inflammation and auto-immune conditions like small cell vasculitis and systemic lupus erythematosus [132]. Proteins derived from NETs may serve as self-antigens and mediate organ damage in auto-immune diseases [133]. Activated neutrophils exert cytotoxic effect on cancer cells by the release of defensins proteins. G-CSF that is produced by most types of cancer cells activates neutrophils and stimulates them to NET formation, whereas some NET components like myeloperoxidase, proteinases and histones, can have a cytotoxic impact on cancer cells and inhibit cancer growth.

The functional relevance of this novel cell-death process by neutrophils was first successfully demonstrated by Brinkmann et al. (2004) [26]. Isolated neutrophils were stimulated by PMA, lipopolysaccharides (LPS) and CXCL8 in vitro and showed as a potent neutrophil activator. These activators led the production of NETs and bacterial killing was observed by these structures. The formation of NETs starts within 10–15 min after the cellular activation and onset of signalling including reactive oxygen species (ROS) generation, activation of kinases and various transcription factors [26,132]. NET formation has been reported in cows, mice, cats, chickens, horses, fish, rabbits and humans [26,139,140,141]. NETs can expand up to 15 times the size of the cells from which it was originated [11]. This tremendously increases the range of effective capture of various small and large sized pathogens and also their subsequent killing or neutralization by the toxic proteins coated on NETs [142]. NETs are membrane free structure and do not carry cytoplasmic proteins like actin, annexin-I, microtubules. These are fragile and smooth fibers with the potential to aggregate into the thick fiber bundles of measuring 50 nm diameter [26,143].

NET components act as alarm signals to activate other immune cells and thus propagate the inflammatory response. The various components of NETs such as DNA and proteins are sensed by the macrophages and dendritic cells which lead them to produce pro-inflammatory mediators. Platelets are also involved in the formation of ETs (extracellular traps). When activated, platelets bind to neutrophils through TLR4 receptor and facilitate network formation]. Platelets, by aggregating to NETs, influence its functioning and enlarge the trap with erythrocytes and other serum factors, like von Willebrand factor, fibronectin, fibrinogen, which stabilizes the NET [91].

NETs play an important role in controlling the extracellular infections. NETs display effectiveness against diverse pathogens including Gram-positive and Gram-negative bacteria, fungi, parasites, and viruses [144]. NETs dis-arm pathogens by sticky DNA mesh with antimicrobial proteins that capture and bind, to kill pathogens extracellularly independent of phagocytic uptake [145]. NETs also prevent the collateral damages of tissues adjacent to the site of inflammation by keeping the potentially injurious proteins like proteases from diffusing away and inducing damage in tissue. NETs may also serve as a physical barrier that prevents further spread of the pathogens. NETs components including IL-37 can promote the inflammatory response through neutrophil recruitment [120]. It may also contribute to persistent neutrophilia as well as pathology of COPD directly through cytotoxic nature and indirectly through inflammation [14]. NETs were initially found to immunoregulate the host defense responses during infections, however, emerging data indicate that delayed NET clearance and/or dysregulated production cause a range of human inflammatory diseases that may lead to tissue damage and organ dysfunction independent of infections [28,146]. Therefore, understanding of the regulatory mechanism involved in NETs release is important to balance NETs role in immunoregulation or disease exacerbations. Next section highlights the known regulatory mechanism of NETs release in infection and non-infectious situations. Major mediators and components of NETs are shown in Table 2.

### 4.1. NETosis: The Formation of NETs

**Figure 2:**
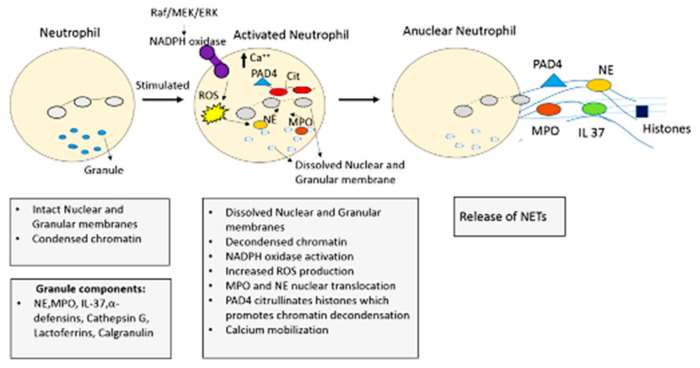
Basic Steps of NETs formation. Micro-organisms, PMA, LPS, endogenous DAMPs and auto-antibodies stimulate the neutrophils to release NETs through several signaling cascades and effector proteins. Activation of the NADPH oxidase via PKC and Raf/MEK/ERK signaling drives the generation of ROS and activation of Peptidyl arginine deaminase (PAD4), which citrullinates arginine on histones leading to chromatin decondensation. Then, NE and MPO are released from azurophilic granules and translocate to the nucleus to promote further chromatin decondensation. Nuclear Envelope breaks down releasing chromatin in cytosol and mixes with cytosolic proteins. Finally, NETs are released.

The cascade of events leading to formation of NETs is termed as NETosis. This process can be initiated by many triggers, for example; the direct stimulation by microorganisms including bacteria, fungi, viruses and pro-inflammatory cytokines and it also appears to be dependent on TLR pathways [134,147,148]. The basic regulatory steps leading to formation of NETs are illustrated in below illustration Figure 2. “NETosis” is the commonly used term for the formation of NETs, but has concerns about its appropriateness to use with, in the light of recent findings. During the formation of NETs whether cell is alive or dead, that is important to define NETs release process. There are evidences that showed that NETs can be produced in the absence of cell death. The Nomenclature Committee on Cell Death (NCCD) in 2018 recommends that the term “NETosis” can be used, in the evidence of cell death. Though there are many evidences where NETosis has not been characterized as recommended by NCCD.

### 4.2. NETosis Mechanism

Understanding of the NETs release mechanism is needed to better discuss the NETs role in the context of disease including COPD. So far, the regulation of NETs formation has been characterized in two ways based on the involvement of ROS, different MAP kinases, calcium influx and granular enzymes, as detailed in subsequent sub-section.

NET formation has been observed in airway fluids of patients with COPD, cystic fibrosis, acute respiratory infection and primary graft dysfunction after lung transplantation [28,149,150,151,152]. Though neutrophils are transcriptionally active cells, most of their DNA is transcriptionally inactive. It is condensed into heterochromatin within the nucleus. DNA is wrapped around the histones to form nucleosomes and further it is organized into chromatin [153]. Peptidyl arginine deaminase (PAD4) catalyzes conversion of histone arginine into citrulline which reduces strong positive charge of histones and the histone DNA binding becomes weak [116]. Due to this weak interaction, nucleosomes are unwrapped, which is a pre-requisite for NET formation [153,154]. NE cleaves the histones during NET formation hence playing and important role in NETosis. Study by Zabieglo et al. reported that secretory leukocyte peptidase inhibitor, which is an endogenous inhibitor of elastase and cathepsin G inhibits NETs formation [155]. Elastase deficient mice were unable to undergo NETosis [156,157].

#### 4.2.1. NADPH Oxidase (NOX) Dependent NETosis

When neutrophils are stimulated by NETotic inducers such as lipopolysaccharide (LPS) or polymethylmethacrylate (PMA), Nox-dependent NET formation is induced [137]. Studies have reported that these agonists (LPS and PMA) induce Nox-dependent NET formation through two different mechanisms. The most potent stimulator of NETosis is PMA [158]. It relies on the activation of p38, Mitogen activated protein kinases (MAPK) and ERK1/2 signaling pathways [135]. After the entry of PMA, endoplasmic reticulum sources of calcium enter the cytosol. This leads to increased activity of protein kinase C (PKC), which in turn phosphorylates gp91phox/Nox2 [138,159]. This process facilitates the assembly of the Nox enzyme, thereby driving the generation of ROS. Intracellular ROS levels are increased due to activation of NADPH oxidase (NOX), which rapidly generates superoxide and H_2_O_2_ by catalyzing electron transfer from NADPH to oxygen. ROS disintegrate the membranes of the nuclear envelope and granules thus allowing the fusion of the DNA released with the granular and cytoplasmic contents.

During NOX-derived ROS production optimal environment is created for NE and MPO [137]. These enzymes which are normally contained within azurophilic granules are now free to interact with the nucleus where they may cleave histones and facilitate chromatin decondensation [160]. These results in loss of membrane integrity of the neutrophil and the decondensed DNA which is decorated with granular contents are released into the extracellular milieu to carry out anti-microbial functions [143].

A separate pathway mediated by c-Jun N-terminal kinases (JNK) functions in LPS-induced Nox-dependent NET formation. Khan et al. reported that although the mechanism is similar to PMA, but there are significant differences upstream of step of phosphorylation of Nox2. Dose-dependent Nox-dependent NET formation is induced by LPS binding to TLR4 on the neutrophil surface [134].

The role of PAD4 in Nox dependent NET formation is controversial. Studies by Ravindran et al. and Douda et al. reported that PAD4 is required for Nox-independent NET formation but does not play an integral role in Nox-dependent NET formation [121,138]. Khan and Palaniyar reported that transcriptional firing is required for NETosis to occur [132]. Different kinases such as Erk, Akt, p38, and cSrc-regulated genes are primarily transcribed during Nox-dependent NETosis [121,137].

#### 4.2.2. NADPH Oxidase (NOX) Independent NETosis

An influx of extracellular calcium through calcium ionophores such as ionomycin and A32178 is required for inducing NOX-independent NETosis [121,161]. Calcium ionophores induce mitochondrial ROS production in a Nox-independent manner [121]. PAD4 in large amounts are already present in the cytosol and binds with the calcium (from the influx provided by ionophores), and translocate into the neutrophil nucleus. PAD4 deiminates histone arginine residues carrying a positive charge into neutral citrulline which results in chromatin decondensation [136,162]. This step is necessary for NOX independent NETosis to take place. Citrullination of histone at promoter sites provides access to transcription factors. The relevance of citrullination of histones in NET formation was studied [121]. Extensive citrullination of histone H3 occurs during Nox-independent NETosis, but not in Nox-dependent NETosis [121]. In study performed by De Souza et al., it was reported that a Nox-independent NET formation agonist (A23187), induces histone H3 citrullination while a Nox-dependent NET formation agonist (PMA), does not induce histone H3 citrullination.

Ca^2+^ ionophore induced NETosis depends on mitochondrial ROS production. Mitochondria in neutrophils serve as a ROS generator and also play a role in facilitating the innate immune function of neutrophils via Nox-independent NET formation [138]. SK3 is the most commonly expressed channel of small conductance (SK) channel on neutrophils. The necessity of the calcium activated potassium channel of small conductance (SK) and mitochondrial ROS (mROS) in Nox-independent NET formation was studied by Douda et al. (2015) [121,163]. Reduction in Nox-independent NET formation following SK3 knockdown and induction following treatment with SK channel-specific activator, 1-Ethyl2-benzimidazolinone (EBIO) was reported [121]. Moreover, studies reported that on treating neutrophil with mitochondrial uncouplers, NOX-independent NETosis was inhibited. Study by Ravindran et al. reported inhibited mROS production leading to a significant and dose-dependent reduction in Nox-independent NET formation on incubation with dinitrophenol (DNP), a mitochondrial ATP production uncoupler [138].

Transcriptome analyses have shown that DNA transcription at multiple chromosome loci during chromatin decondensation occurs faster in the NOX-independent form than in the NOX-dependent form [137]. The transcription of *Akt*, *p38*, *cSrc*, *PyK2* and *Jnk* regulated genes occur mainly in Nox-independent NETosis. Low levels of ERK and moderate levels of Akt activation were reported in NOX-independent NETosis, as compared to NOX-dependent NETosis [137].

## 5. NETs and COPD

**Figure 3:**
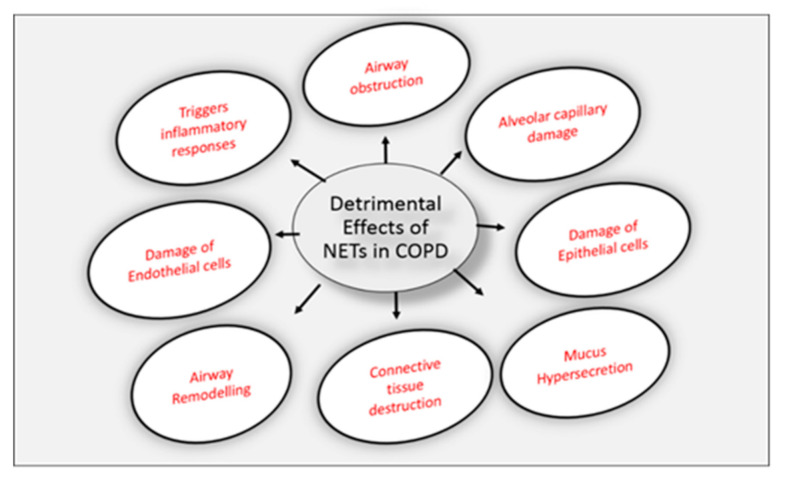
Detrimental effects of NETs in COPD: Inflammatory responses are triggered by elevated NETs in COPD and are associated with disease severity leading to airway obstruction, remodeling and mucus hypersecretion. NETs can also cause lung tissue damage by causing destruction of connective tissues and it also causes damage of alveolar capillaries, epithelial and endothelial cells.

NET formation in both stable and exacerbated COPD patients was reported for the first time using confocal fluorescent and electron microscopy, in 2015 [149,164]. Varying degrees of damage to lung tissues is caused by dysregulated excess production of NETs in the airways or lung tissue. Death of human epithelial and endothelial cells can be induced by the prolong presence of NETs, thus resulting in impaired pulmonary function. This can also lead to progression of the disease [165]. NET formation can lead to many indirect complications besides causing direct tissue damage. Airway obstruction caused by thick sticky DNA and protein rich mucus plugs is a pathological hall mark of COPD. The extracellular DNA content present in the mucus plugs of patients were re-analyzed and it was found to be from neutrophilic origin deposited in NETs [166,167]. The high extracellular DNA content enhances the viscosity of mucus [117]. Thus, the extensive NET formation during chronic respiratory diseases can contribute to airway-obstruction. The detrimental effects caused by NETs in COPD are depicted in Figure 3.

The genesis and structure of NETs was studied by Obermayer et al. from sputum of COPD patients [150]. Sputum of patients of all grades of COPD, stable and exacerbated are characterized by presence of large amounts of NETs, NET forming neutrophils and increased degranulation has also been seen in the neutrophils from COPD patients. Upregulation of NET formation in COPD patients was observed by Pederson et al. which was associated with higher concentrations of extracellular DNA in sputum supernatant of these patients [164]. The level of extracellular DNA/NETs was also found to be inversely correlated with lung-function [168] and this NET abundance was also shown to be correlated with the degree of airflow limitation as measured by FEV_1_ [149] and exacerbation frequency [25]. It was found that in stable COPD patients, the NET formation by sputum neutrophils and extracellular DNA levels in sputum supernatant was found to be significantly increased irrespective of the current smoking status and purulence of the sputum sample [164]. Translational studies were conducted using confocal fluorescent and electron microscopy by Pederson et al. and Grabcanovic- Musija et al. respectively [149,164]. These authors first observed NET formation in sputum from both stable and exacerbated COPD patients. The presence of large amounts of NETs is associated with disease severity as it was present in over 90% of exacerbated COPD patients as compared to 45% of stable COPD patients [149]. NET formation was reported in sputum of COPD patients hospitalized for an acute exacerbation in a study by Obermayer et al. (2014) [150]. NET clearance by DNase’s is impaired in COPD patients. Study performed by Dicker et al. showed a correlation between NET complexes and microbial diversity in COPD sputum samples [25]. Thus, NETs are more abundant in severe COPD patients and are associated with more frequent exacerbations and reduced microbiota diversity. Increased NET production following LPS stimulation also occurs in peripheral blood derived neutrophils of stable COPD patients [8]. Activation of PAD4 is an important regulator of NET formation [169]. *PAD4* gene expression was upregulated in neutrophilic COPD patients and elevated NET formation was also associated with lung functions and COPD symptoms [8,168]. Increased expression of *PAD4* in lungs of COPD patients at protein levels was reported by Lugli et al. (2015) [170]. It is not yet clearly established whether the disease state or excess NETs is responsible for the disease exacerbation. Focused studies needed to understand the effector and cause relation between NETs and COPD disease progression or exacerbation.

The mechanism of NET formation in COPD still remains to be explored. It is not clear whether neutrophils undergo NETosis following migration into the lung tissues or whether neutrophils are constitutively poised to undergo this response in circulation during COPD related inflammation. The active constituents of NETs including cell-free DNA and MPO were also reported to be increased in peripheral blood of COPD patients [25]. NETs also have a role in amplification and perpetuation of inflammation. It can promote inflammation through NOD, LRR- and pyrin domain-containing protein 3 (NLRP3) inflammasome and neutrophil chemotaxis [171]. The NET components IL-37 and α-defensin are both able to induce inflammasome activation [172]. IL-37 can drive CXCL8 release from epithelial and smooth muscle cells leading to positive feedback for further neutrophilia. The NETs would have been degraded by endogenous nucleases and also cleared by alveolar macrophages under physiological conditions [173]. Lower numbers of alveolar macrophages are present in neutrophilic COPD patients and these macrophages are defective in phagocytosis [174], which may explain the abnormal persistence of NETs in the airways. A Study by Nakazawa et al., focused on the interaction of macrophages and neutrophils that underwent NETosis. Macrophages displayed a phenotype-dependent response after degradation of NETs. Several hours after the interaction, M2 macrophages induced a pro-inflammatory response and hence helped to perpetuate the inflammatory response. M1 macrophages underwent cell death with nuclear decondensation which took place in a PAD4 dependent manner and resulted in a local release of extracellular DNA. Thereafter, M1 macrophages degraded DNA derived from themselves in a caspase-activated DNase-dependent manner resulting in the clearance of extracellular DNA within 24 h. This suggests a phenotype-dependent mechanism of macrophage regulation of NETs release [175]. Previous reports indicating the relationship between smoking and NET formation are contradictory. Studies suggests that NETs can be induced by nicotine which is the addictive component of tobacco and NETosis induced lung injury might have occurred in smokers without airflow limitation [176]. However, other reports suggest that current smoking status of COPD patients does not affect NET formation [164].

## 6. Role of CXCR1 and CXCR2 Receptors in Neutrophils and COPD

Various mediators such as CXCL8, IL-1B, TNF-α and LTB4 are involved in neutrophil recruitment to the airways [177]. The inflammatory cytokines achieve their effects by binding to CXCR1 and CXCR2 receptors located on neutrophils cell membrane. The ligand receptor relationships are rarely exclusive, thus there is extensive functional redundancy in signaling pathways [178]. In COPD airway secretions, CXCL8 is the predominant neutrophil attracting chemokine which accounts for trafficking of approximately 1/3rd of neutrophilic infiltrates in sputum [7]. CXCL8 signals through both the receptors, CXCR1 and CXCR2 [178] which have similar signaling mechanisms like phosphoinositide hydrolysis, intracellular Calcium mobilization and chemotaxis. However, CXCR1 and CXCR2 might have different physiological roles under inflammatory conditions. CXCR1 has a role to play specifically in phospholipase- D activation, respiratory burst activity and bacterial killing by neutrophils [179]. It is mainly responsible for degranulation of neutrophils while recruitment of neutrophils from blood to tissues is regulated by CXCR2. It is a receptor for number of chemokines such CXCL1-3 and CXCL8 all of which are reported to be elevated in COPD [180]. In severe COPD exacerbation patients, increased expression of both CXCR1 and CXCR2 was seen in endo bronchial biopsies [181]. NET formation is regulated by chemokine receptor CXCR2 in COPD neutrophils invitro [8].

### CXCR2 Inhibition in COPD and NETs

For the NETopathic inflammation occurring in COPD, NET stabilizing therapies via CXCR2 blockade could be leveraged. Since CXCR2 plays a homeostatic role in regulating neutrophil egress from bone-marrow to blood [182], it is expected that targeting CXCR2 would reduce neutrophilic inflammation, mucus production and neutrophil-proteinase mediated tissue destruction in lungs [178]. In a translational study by Pederson et al., the levels of NET formation in blood and sputum neutrophils of COPD patients were compared ex-vivo. NET formation, extracellular DNA and concentration of CXCL8 was studied after incubation with sputum supernatant derived from the same COPD patient in an autologous manner in the presence and absence of CXCR2 antagonist (AZD5069). It was observed that spontaneous NET formation was absent in peripheral blood neutrophils. However, significant NET formation was observed upon stimulation with sputum supernatant of the same individual, compared to naïve, unstimulated blood neutrophils, NET formation was increased by five-fold when blood neutrophils were stimulated with sputum supernatant. Extracellular DNA and CXCL8 concentration were significantly higher in stimulated blood neutrophils compared to unstimulated blood neutrophils. Thus, this study reported that neutrophils from COPD patients are likely to undergo NETosis upon exposure to inflammatory microenvironments conducive to the airways of COPD patients. CXCR2 antagonist AZD5069 reduces NET areas and DNA release by blood neutrophils stimulated by autologous COPD sputum supernatant. Thus, CXCR2 has an essential role in regulating NETosis via CXCL8-mediated mechanism in neutrophils derived from blood of COPD patients. Sputum neutrophils showed spontaneous NET formation and this response was significantly decreased in the presence of AZD5069. The effect of CXCR2 antagonist on spontaneous NETosis of sputum neutrophils was less. This suggests that neutrophils that have already undergone NETosis, their reversal via CXCR2 antagonist cannot be achieved [164].

Study conducted by Holz et al., indicated that treatment with SCH527123, a selective CXCR2 inhibitor significantly decreased ozone induced airway neutrophilia in induced sputum of healthy subjects. Moreover, the levels of CXCL8 and MPO were also found to be significantly decreased by SCH527123. Thus, these results supported the hypothesis that CXCR2 inhibition may have beneficial role in COPD [183]. Significant reduction in severity of dyspnea in COPD patients as compared to placebo was reported in previous studies by using monoclonal antibodies against CXCL8 and ABX-CXCL8 [184]. In a recent clinical study performed by Rennard et al., use of CXCR2 antagonist (MK-7123) in COPD patients has shown a significant neutrophil lowering effect leading to improvements in FEV_1_ and reductions in exacerbations in active smokers as compared to placebo [185]. Clinical trials are undergoing to evaluate the modulatory role of CXCR2 antagonists in sputum NET production in COPD patients. (Clinical Trials. Gov- Identifier: NCT03250689). Previous studies reported elevated levels of PGP (proline-glycine-proline), which is an extracellular matrix derived fragment in the airways of COPD patients in response to cigarette smoke [186]. It contains reactive aldehyde and tussive agent acrolein which can directly induce NET production [187]. Thus, during COPD related airway inflammation, PGP could also trigger NET formation. There occurs PGP-CXCR2 cross talk in COPD patients. Potent CXCR2 signaling ligands such as acetyl-proline-glycine-proline (AcPGP) and PGP fragments are released by acrolein. CXCR2 signaling thus regulates NET production in COPD neutrophils. Thus, CXCR2 inhibitors could be used as a potential anti-inflammatory therapy in diseases with chronic neutrophilic airway inflammation such as COPD [8]. Hence it is believed that blocking of CXCR2 receptor can prevent the neutrophils from migrating into the airways [188] and thus, will be unable to release their inflammatory proteinases into the airways hence preventing the progression of COPD. CXCR2 antagonists have been developed to selectively block neutrophilic inflammatory pathways. This could lead to novel therapeutic strategies for multiple CXCR2 related NETopathologies.

## 7. Vicious Cycle of the NETs in COPD Inflammation

An important part of COPD inflammatory process is the activation and aggregation of neutrophils in the lung [65]. Recurrent bacterial and viral infections are the main causes of exacerbations in COPD patients and are associated with increased upper and lower airway and systemic inflammation [189]. Thus, the trigger for neutrophilic inflammation is colonization of microbiota in the airways. Severe COPD patients present large number of airway neutrophils when they are stable, and these numbers further increases during exacerbations. This may be due to the high expression of neutrophil chemokines and chemokine receptors in airway mucosa [3]. Thus, there is an excessive infiltration and activation of neutrophils, production of ROS and release of serine proteases such as MMPs and MPO, resulting in collateral damage as the cells infiltrate into the tissues [14]. Due to dysregulated apoptosis, increased neutrophil survival occurs, which facilitates continued release of neutrophil derived mediators to perpetuate airway inflammation and tissue injury [190]. Chronic airway mucus hypersecretion and the destruction of the lung parenchyma is induced by these neutrophils through the release of NE which plays a pro-inflammatory role in COPD by stimulating the secretion of CXCL8 [3]. It is noteworthy that CXCL8 is a potent NET inducer [26]. Thus, COPD is a prominent candidate for NETs formation and NETosis-mediated tissue damage.

It is assumed that in COPD patients, NETs are responsible for the chronic inflammatory condition and lung function decline. However, the pathophysiology of NETs involved in the airway’s inflammation and lung injury in COPD patients remains unclear. NETs might be directly cytotoxic to airway epithelial and endothelial cells, as they contain a mixture of extracellular DNA, histones, and granular proteins [191]. These NETs may also indirectly induce injury to the lung tissue through the promotion of autoimmune reactions against an aberrant amount of NETs components [192]. Levels of NETs and NET components in COPD are associated with markers of innate immune responses such as IL-1β and CXCL8 and NOD-like receptor family, pyrin domain containing 3 which is an inflammasomes component [168]. Thus, the positive feedback of pro-inflammatory cytokines and neutrophilic chemokines is responsible for persistent airway neutrophilia seen in COPD. This also promotes the production of additional NETs, thereby creating a vicious cycle [14].

### 7.1. Future Prospects: Targeting NETs in COPD

Current treatment for COPD involves the use of long-acting bronchodilators and is currently the most successful, but no therapy reduces the progression or inhibits the inflammation in COPD patients [35]. There has been substantial research regarding the antibacterial properties of NETs. It has been widely accepted that NETs play an essential role in trapping and killing microbes to prevent microbial dissemination. Although NETs play an essential role in the innate immune system against infection, the excessive NETs in the airways or lung tissue of COPD patients can cause varying degrees of damage to the lung, resulting in impaired pulmonary function and the acceleration of disease progression in COPD patients [14,168]. The severity of COPD patient was also shown to be positively correlated with the level of NETs in their airways [149]. Fine-tuning of NET formation throughout the course of the chronic inflammatory diseases is the goal for the development of novel NET-targeted therapies. Selective inhibitors of NET formation or NET associated proteins such as NE, MPO, histones may prove valuable as NETs are implicated in disease worsening [15]. Thus, inhibiting NET formation is an attractive strategy for preventing the deleterious effects of NETs or their components in COPD patients. It is yet to be explored whether directly targeting NETs or regulating neutrophil activation can inhibit the development of disease in COPD patients. Previous studies indicated that the harmful effects of neutrophils on the lung tissue can be limited by using CXCR2 antagonists as it can reduce neutrophils in the lungs of COPD patients [193]. Therefore, by targeting NETs, we can develop future strategies to regulate neutrophil influx and function. This can be achieved by elucidating the regulation of NETosis, understanding the functioning of NETs in these chronic inflammatory diseases, and increasing our understanding of the molecular mechanisms behind NET formation. This will prove to be helpful for developing novel potential therapeutic targets and customizing treatment for chronic inflammatory airway diseases in the future.

### 7.2. Potential Anti-Net Therapeutics

Targeting key factors involved during the formation of NETs, NETs integrity, and specific proteins of NETs will promise novel therapeutic strategies.

**Figure 4:**
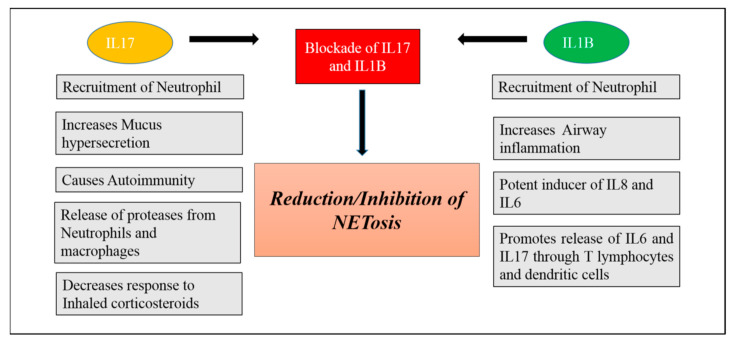
Flow chart showing the potential Anti-NET Therapeutics targets by blockade of IL17 and IL1B.

1. Anti-thrombosis: Heparin is a naturally occurring glycosaminoglycan which is used as an anticoagulant (blood thinner) in the treatment of stable angina, heart attacks and it also antagonizes the effects of histones [194,195,196]. Role of Heparin in reduction/inhibition of NETs is enlisted in Table 3.
2. Nucleases: DNAses acts on DNA Matrixes and cleave it and reduces the infiltration of neutrophils hence playing role in inhibition/reduction of NETs (Table 3) [196,197].
3. NADPH/ROS inhibitor: Hydroxychloroquine (HDQ) is a less potent derivative of chloroquine. It is an anti-malarial drug used to treat malaria. HDQ mediated MMPs-TIMPs interaction helps in maintaining homeostasis of extracellular matrix and hence may play role in reducing NETs (Table 3) [196,198].
4. Blockade of IL-1B and IL17: IL1B and IL-17 are the key mediators of neutrophilic airway inflammation in COPD. Elevated serum levels of these cytokines may be used as a bio-marker for indicating persistent neutrophilic airway inflammation and potential ongoing exacerbation in COPD. Levels of these two inflammatory mediators in serum are associated with important clinical parameters in COPD such as degree of airflow limitation and smoking status. As we know that neutrophils can produce and release NETs in COPD, these NETs in turn can prime macrophages to produce a precursor form of inflammatory cytokine IL-1B (pro- IL1B). NETs can also collaborate with other activation signals such as heat shock proteins and cholesterol signals promoting the release of IL-1B [199]. Thus, serum IL-1B increase in COPD is also found to be associated with neutrophil percentage in COPD. Thus, neutrophils mediate formation of IL-1B that facilitates neutrophil recruitment into airways creating a vicious cycle of neutrophilic airway inflammation and contributing to progression of COPD. IL17 is mainly secreted by IL17 producing T lymphocytes including αβ T cells and ϒ𝛿 T cells and these two kinds of cells can be induced by IL1B in the lung tissue and BALF of COPD patients [200]. Thus, IL1B may be an important factor leading to increased expression of IL17 in COPD. Blockade of IL1B and IL17 could be a valid strategy for prevention and control of COPD [201,202] (Figure 4).

## 8. Conclusions

The paradigm of neutrophil-mediated innate immunity has been reshaped by the identification of NETs. Though NET formation is an effective antimicrobial defense strategy, yet its dysregulation may impart bystander consequences and hence contribute to NETopathic inflammation. Studies targeting direct interruption of NET functioning or strategies to regulate neutrophil activation in COPD patients are desperately needed. A novel approach to treat COPD patients could be through inhibition and regulation of NETs. Stabilizing the aggressive potential of NETs to homeostatic control should be aimed rather than completely neutralizing this process. As no curative therapy is currently available for COPD, we anticipate that disentangling the NETopathic inflammation pathways could lead to the development of innovative therapeutics for COPD.

## Figures and Tables

**Table 1:** Factors that determine neutrophils decision to phagocytosis or form NETs.

Determining Factors	Phagocytosis	Formation of NETs	References
Integrity of cytoskeleton	Cytoskeleton integrity is required for phagocytosis	Cytoskeleton disruption is a pre-requisite for NETs formation	[90,107]
Size of pathogens	Small size of pathogens favors phagocytosis	NETs released in response to large pathogens	[27,90]
Key signals required	MPO is not required	NE and MPO are the key granular proteins which get activated during generation of NETs	[116,117]
Immunoglobulin(Ig) opsonization	Ig opsonization required	Ig opsonization is not required	[90,106]
Autophagy	PS recognition leads to phagocytosis	Autophagy induced by PMA	[112,113,114,115]
Role of platelets: High mobility group box-1 (HMGB1)	HMGB1 is known to be an effective inhibitor of phagocytosis	Neutrophils are instructed by platelets through HMGB1to release NETs via a pathway that involves HMGB1 receptor	[110,111]
Role of pH	Phagocytosis of opsonized bulky particulates is ensured by an acute drop in intracellular pH	Acidic environments impair NET formation	[108,109]
Role of DEK	DEK is not required for phagocytosis	DEK is necessary for NET generation	[90,105]

**Table 2:** Major mediators and components of NETs.

Compartments	Mediator and Components of NETs	References
Cytoplasmic	Calprotectin and Catalase PAD4 (mediating citrullination of histone3; CitH3) Kinases; ERK, Akt, JNK, p38, Src etc.	[125,134,135,136]
Granular	Primary granules (e.g., MPO, cathepsin G and neutrophil elastase) Secondary granules (e.g., lactoferrin and pentraxin 3) Tertiary granules (e.g., gelatinase and peptidoglycan binding protein)	[124,135,137,138]
Nuclear	DNA and Histones (H1, H2A, H2B, H3, and H4) Activation of transcription factors (transcriptional firing) Citrullination of histone by PAD4 (CitH3) Activation of Gasdermin-D to make pores	[26,121,122,137,138]

**Table 3:** Potential Anti-NET Therapeutics: Heparin, DNAse and Hydroxychloroquine.

Compartments	Mediator and Components of NETs	References
Heparin	Interferes with neutrophil autophagy Suppresses Histones Prevents platelets-histone interaction Blocks HMGB1	[194,195,196]
DNAses	Reduces neutrophil infilteration Hydrolyzes DNA Reduces viscosity in lungs	[196,197]
Hydroxychloroquine	Targets endosomal NADPH oxidase Inhibits cytokine production Maintains extracellular homeostasis	[196,198]
